# Organ-specific immune-mediated reactions to polyethylene glycol and polysorbate excipients: three case reports

**DOI:** 10.3389/fphar.2023.1293294

**Published:** 2024-01-03

**Authors:** Olga Rogozina, Carmen Ruiz-Fernández, Susana Martín-López, Ibtissam Akatbach-Bousaid, Miguel González-Muñoz, Elena Ramírez

**Affiliations:** ^1^ Clinical Pharmacology Department, La Paz University Hospital-IdiPAZ, Faculty of Medicine, Autonomous University of Madrid, Madrid, Spain; ^2^ Immunology Department, La Paz University Hospital-IdiPAZ, Madrid, Spain

**Keywords:** case report, adverse drug reactions, delayed hypersensitivity reactions, causality algorithms, lymphocyte transformation test, excipients, CD69 upregulation

## Abstract

Drug-related acute pancreatitis (AP), acute interstitial nephritis (AIN) and drug-induced liver injury (DILI) are rare but serious adverse drug reactions (ADRs) that can have life-threatening consequences. Although the diagnosis of these ADRs can be challenging, causality algorithms and the lymphocyte transformation test (LTT) can be employed to help with the diagnosis. In this report, we present 3 cases of drug-related AP, AIN and DILI. The first case involved a patient with AP to lacosamide and to the excipient polysorbate 80 in pantoprazole. The second case involved a patient with DILI secondary to polyethylene glycol (PEG) excipients and amoxicillin-clavulanate. In case 3, AIN was considered to be the result of sensitization to excipients. Diagnoses were made using causality algorithms and the LTT. The LTT is a useful tool for helping diagnose drug-related AP and DILI, and it can be used to identify the specific drug or excipient causing the ADR. These cases highlight the importance of considering PEG and polysorbate excipients in the causality diagnosis of ADRs.

## Introduction

Drug-related acute pancreatitis (AP), acute interstitial nephritis (AIN) and drug-induced liver injury (DILI) are rare but serious adverse drug reactions (ADRs) that can have life-threatening consequences. The incidence of drug-related AP is estimated at between 0.1% and 2% of all AP cases (Wolfe et al., 2020), and DILI is now recognized as the fourth leading cause of liver damage in Western countries (Katarey and Verma, 2016). Accurately identifying AP etiology is crucial for reducing the morbidity and mortality associated with an AP episode, particularly in patients with recurrent AP. DILI is a rare but significant adverse event associated with numerous pharmaceutical agents, herbal remedies, and dietary supplements. Vigilance among treating physicians in recognizing possible DILI cases is essential for promptly discontinuing the implicated agent and excluding other potential causes. It has been estimated that drug-related AIN is the third most common cause of acute kidney injury in hospitalized patients (Sanchez-Alamo et al., 2023).

The diagnosis of drug-related AP, AIN and DILI can be challenging, given that frequently there are no specific signs or symptoms that definitively point to a drug as the cause. Rechallenge with the suspected drug is not allowed in serious ADRs. The diagnosis is typically based on ruling out other possible causes of the patient’s symptoms, which could involve performing a thorough medical history, physical examination, and laboratory tests. If other causes can be ruled out, then the ADR is more likely to be caused by a drug. Applying causality algorithms to the suspected drugs when patients have been exposed to several drugs can help identifying the causal drug/s.

Causality algorithms are structured and standardized scales used to quantify the association between a drug and an ADR. These algorithms can be used to help make a diagnosis of serious ADRs, but they have the limitation that they can be difficult to use when multiple drugs are taken at the same time (Macedo et al., 2006). Some *in vitro* testing can help a clinician in the diagnostic process.

The lymphocyte transformation test (LTT) is a laboratory test that can be used to help diagnose serious ADRs. The LTT measures the response of white blood cells (lymphocytes) to a drug that is suspected of causing the ADR. If the lymphocytes show a significant increase in response to the drug, then this is a strong indication that the drug is the cause of the ADR (Rodri et al., 2022). Another approach is the flow-cytometry analysis of drug-induced CD69 upregulation in T cells (Beeler et al., 2008). Detailed information on causality algorithms and methodological aspects of LTT and flow cytometry is provided in Supplementary Methodology.

Excipients are inactive ingredients added to medicines to help improve their stability, safety and efficacy. Polyethylene glycol (PEG) and polysorbate (PS) 80 are common excipients used in medications. They are both non-ionic surfactants; they do not dissolve in water but form micelles, or small spheres, that can help to solubilize other substances. Absorption, distribution, metabolism, and excretion of different sized PEG polymers and PS80 depends on many factors, such as size and route of administration (European Medicine Agency; Yin et al., 2022).

They can cause adverse reactions in some people, but these reactions are typically mild and resolve on their own. In rare cases, however, they can be more serious, even causing gastrointestinal, liver, kidney, or neurological damage (Wenande and Garvey, 2016).

The authors present three case reports of patients who experienced adverse reactions to alcohol excipients in medicines. One case involved a patient with AP to lacosamide and to the excipient PS 80 in pantoprazole, another involving a patient with DILI secondary to excipients of PEG and amoxicillin-clavulanate (AMX-CLV) and an AIN case considered to be the result of sensitization to excipients. We have summarized the characteristics of the ADR cases and *in vitro* testing results in Table 1.

**TABLE 1:** Characteristics of ADR cases and *in vitro* testing results.

Case	Sex/Age (years)	Adverse reaction	Suspected drugs^a^ (algorithm score)	LTT	Excipients	LTT
1	M/41	Pancreatitis	Lacosamide (SEFV +5)	Lacosamide: +		
			Valproic acid (SEFV +4)	Valproic acid: −	PEG 3350	-
			Pantoprazole (SEFV +4)	Pantoprazole (b): −	PS80	+
				Pantoprazole (g): +		
2	F/77	Hepatitis	Amoxicillin/Clavulanic (RUCAM 7)	Amoxicillin/Clavulanic: −	PEG 3350	+
					PEG 4000	+
					PS80	-
3	F/81	Interstitial nephritis	Omeprazole (SEFV +5)	Omeprazole: −	PEG 3350	+
			Metformine (SEFV +2)	Metformine: −	PS80	+

b, brand-name drug; g, generic drug; PEG, polyethylene glycol; PS, polysorbate.

^a^ Algorithms used in cases 1 and 3 was SEFV (Yin et al., 2022) and RUCAM (Wenande and Garvey, 2016) for case 2.

Informed consent was obtained from the patients before publishing. A complete adverse reaction report for each case was submitted to the Spanish National Health Authorities (Pharmacovigilance Center in Madrid), with case numbers NR66287, NR64097, and NR66197 respectively.

## Cases description

### Case report 1

A 41-year-old male patient with a medical history of drug-resistant epilepsy since 2016 and AP due to cholelithiasis with subsequent cholecystectomy was urgently admitted to the Digestive System Department of the hospital with AP diagnosis. The patient denied alcohol consumption, toxic substance abuse, family history of pancreatic diseases, unexplained weight loss or new onset of diabetes. He actively smokes tobacco, 15 cigarettes daily. Before hospitalization, the patient had been prescribed valproic acid 2,400 mg daily during 6 years, lacosamide 600 mg daily during 4 years, clobazam 30 mg daily during 2 years, folic acid 5 mg daily during 6 years and pantoprazole (brand-name [bn]) 40 mg daily during 6 years. Major interactions between drugs taken by the patient were not found (Leigh Ann Anderson). The patient had had previous pharmacological adverse reactions: drug reaction with eosinophilia and systemic symptoms due to eslicarbazepine, behavioral disturbances due to levetiracetam and psychiatric symptoms due to perampanel.

During admission, a physical examination and laboratory and radiology tests were performed to establish the etiology. The biochemical test revealed an elevation of pancreatic enzymes: amylase 259 UI/L (upper limit of normality [ULN], 118), and lipase 292 UI/L (ULN, 53); whereas the liver test (alanine aminotransferase [ALT], aspartate aminotransferase [AST], gamma-glutamyl transpeptidase [γ-GT], alkaline phosphatase), bilirubin and triglycerides remained consistently normal. Also, IgG4, calcium, glycemia, coagulation status and albumin were obtained to identify the cause, and the results were under the ULN.

In contrast-enhanced abdominal computed tomography, realized on the first day of admission, diffuse enlargement of the pancreas with necrosis of pancreatic tissue <30% was detected. No complications were detected in the bile duct. Findings of the abdominal ultrasound performed on day 3 revealed previous cholecystectomy with no visualization of the pancreas.

According to the clinical history and the results of the laboratory and radiology tests, a pharmacological etiology of pancreatitis was suspected. Despite valproic acid levels being within the therapeutic range (between 50 and 100 μg/mL), the dose was reduced to 2,000 mg daily after a neurology assessment. Despite the patient’s condition improving, the reduced dose was maintained due to adequate through level of valproic acid, an adequate seizure control and the patient’s medically refractory epilepsy. The trough level of valproic acid at admission was 68.03 μg/mL, 7 days after the adjustment was 75 μg/mL. After intravenous hydration, pain control and fasting, the patient presented significant clinical and laboratory improvement within 8 days of hospitalization, was discharged and referred to the pharmacovigilance department to investigate the possible pharmacological etiology of the pancreatitis. The etiology was assessed by employing the causality assessment in reports on adverse drug reactions algorithm of the Spanish pharmacovigilance system (SEFV) (Aguirre et al., 2016). Lacosamide (score + 5), valproic acid (score +4) and pantoprazole (score +4) were the drugs involved according to the algorithm, clobazam was conditional (score +3). Seven months later, an LTT was performed to evaluate drugs with a related causality (SEFV score ≥ +4). The LTT showed an immune response to lacosamide (SI = 4.1), agreeing with the causality algorithm. Lacosamide contains the alcohol excipient PEG 3350, which was tested and did not show any T-cell response (SI < 2). Considering the cross-reactivity, we also tested another alcohol, PS 80, which was able to induce T-cell proliferation (SI = 4.8). The pantoprazole used in LTT was a brand-name form not containing PS 80 or PEG 3350, and it did not trigger T-cell proliferation *in vitro*. Valproic acid was also tested and the result was negative (SI < 2).

After we had communicated the results to the patient, he mentioned that the pantoprazole used in the episode was a generic (g) drug, but he had suspended it due to general discomfort. The patient had kept the drug and was asked to provide a sample to the hospital, because after the excipient revision, we discovered that pantoprazole-g contained PS 80 and PEG 3350.

Ten months after the pancreatitis episode, the second LTT was performed. We repeated the lacosamide test, which remained positive. However, pantoprazole-g induced T-cell proliferation (SI = 5.7), in contrast to pantoprazole-bn (SI < 2).

We concluded that the positive LTT result for pantoprazole-g and negative for pantoprazole-bn and PEG 3350 indicated that the type IV hypersensitivity reaction to pantoprazole-g was due to the presence of PS 80.

Given these results, the clinicians continued with the reduced dosage of valproic acid, and they suspended lacosamide and pantoprazole-g.

### Case report 2

A 77-year old female patient was urgently admitted to the Digestive System Department with a 1-week history of jaundice. Her medical history included past hepatitis B virus infection, dyslipidemia and hypothyroidism, for which she was taking daily atorvastatin 10 mg for 8 years, acetylsalicylic acid 100 mg for 3 years, levothyroxine 88 mg for 4 years and omeprazole 20 mg for 10 years. She had been taking amoxicillin-clavulanate (AMX/CLV-g) 875 mg/125 mg three times per day for 6 days for acute otitis and developed jaundice 1 week later. She had no medical or family history of hepatic disease, and no history of previous allergies, alcohol consumption or drug abuse.

Clinical examination showed an afebrile, icteric patient with epigastric abdominal pain during superficial palpation, with no peritonitis signs. Laboratory tests performed on the first day of admission revealed an elevation of total serum bilirubin to 9 mg/dL (ULN, 1.2), with direct bilirubin of 6.88 mg/dL (ULN, 0.3), ALT 83 U/L (ULN, 35), AST 55 U/L (ULN, 40), serum alkaline phosphatase 267 (ULN, 116) and γ-GT 318 (ULN, 38). Hemoglobin and total leukocyte count were normal, and there were no clinical or biochemical signs of acute liver failure. An abdominal ultrasound performed on the first day of admission demonstrated hepatic steatosis and extrahepatic biliary duct dilatation of 8 mm without findings of bile duct or gallbladder stones. The ultrasound was repeated the following day and did not display the bile duct dilatation but detected a possible gallbladder polyp, and the liver’s echogenic structure was completely normal. Magnetic resonance cholangiopancreatography discovered extrahepatic bile duct ectasia of 7 mm with intraductal papillary mucinous neoplasm of the pancreas, which could explain the ectasia. Endoscopic ultrasound showed 2 pancreatic cysts of 7 mm, extrahepatic biliary duct dilatation of 7 mm and symmetric bile duct wall thickening of 9 mm, which are features of chronic pancreatitis without alarm findings. Hepatitis C and E virus, HIV, immunoglobulin (Ig)M cytomegalovirus and IgM Epstein-Barr virus antibodies were negative. IgG cytomegalovirus, IgG toxoplasma, IgG Epstein-Barr, IgG hepatitis A, total HBV core and HBV surface antibodies were positive.

Considering the above results, the diagnosis of cholestatic DILI secondary to AMX-CLV was established. The case definition of DILI relied on the following clinical chemistry criteria: 1) ALT levels ≥5 times the ULN, 2) alkaline phosphatase (ALP) levels ≥2 times the ULN or 3) ALT levels ≥3 times the ULN and, simultaneously, bilirubin levels >2 times the ULN (Ersoz et al., 2001; Kuna et al., 2018). The pattern of liver injury was determined using R value, where R is defined as (ALT/ULN)/(ALP/ULN), cholestatic DILI is characterized by R ≤ 2 (Aithal et al., 2011). The patient received conservative management with analgesia, hydration and symptom control. She was discharged on the seventh day of admission after significant clinical and laboratory improvement. The allergy to AMX/CLV was registered in her electronic clinical history by the moment of the discharge.

The pharmacovigilance department performed both the admission assessment and subsequently the ambulatory follow-up. The DILI’s drug causality was assessed with the updated Roussel Uclaf Causality Assessment Method (RUCAM 2016) (Danan and Teschke, 2016). According to the RUCAM scale, AMX/CLV-g was the implicated drug with a score of +7, metformine (score +2) was conditionals, and the rest of medication (score ≤ 0) were not related. Ten months later, an LTT was performed that did not show any immune response to AMX-CLV or cross-reactivity to other beta-lactam antibiotics. We decided to perform another LTT with alcohol excipients, inasmuch as AMX/CLV-g contains PEG 4000 in contrast to the AMX/CLV used in LTT. This test, performed 1 month later, showed T-cell proliferative responses to PEG 3350 and PEG 4000, but not to PEG 2000 and PS 80. We performed another LTT to alcohol excipients 1 month later after the second LTT, that is, approximately 1 year after drug exposure, including PEG 6000, given that it is an excipient found in other AMX/CLV forms. The test was negative for all alcohol excipients.

Our recommendations initially were to prohibit amoxicillin, AMX-CLV and all the drugs that contain polyethylene glycol with molecular weight superior to 2000 as an excipient or as an active component. Eventually, after the third LTT, we removed contraindications to alcohols but preserved the prohibition of amoxicillin and AMX-CLV due to our inability to rule out their involvement in DILI development. We believe that both macrogols and AMX-CLV more likely contributed to the liver damage.

During ambulatory follow-up the patient presented complete resolution of the symptoms and biochemical results within 7 months.

### Case report 3

A 79-year-old female patient was urgently admitted to the Nephrology Department of our hospital because of impaired renal function and anemia found on routine laboratory testing. She reported a month-and-a-half history of fatigue, anorexia, involuntary weight loss, occasional nausea, vomiting and abdominal pain.

Her medical history showed dyslipidemia, hypothyroidism, hypertension and type 2 diabetes. The patient’s regular medications at the time of admission were enalapril 30 mg daily, omeprazole 20 mg daily for 6 months, metformin 850 mg daily for 2 years, vitamin D3 0.266 mg monthly for 4 years, simvastatin 20 mg daily for 9 years, levothyroxine 75 mg daily for 8 years and amlodipine 5 mg daily for 9 years. She had no family history of nephropathy, no toxic habits and reported no exposure to any nephrotoxic agents.

Physical examination was unremarkable except for high blood pressure 163/82 mm Hg and pallor. Regarding the laboratory investigations, serum creatinine was 2.37 mg/dL (ULN, 1.1) with an estimated glomerular filtration rate of 20 mL/min/1.73 m^2^. Her baseline eGFR was 84 mL/min/1.73 m2 and serum creatinine 0.66 mg/dL, which were determined 6 months before the admission. She was diagnosed of stage 2 of acute kidney injury according to KDIGO criteria without oliguria. Serum potassium was 5.5 mmol/L (ULN, 5.1), blood urea nitrogen was 109 mg/dL (ULN, 49), hemoglobin was 8.9 g/dL (normal range 11.8–15.8) and platelet and leukocyte counts were normal. Urinalysis showed proteinuria (0.19 g/day) and albuminuria 41 mg/24 h, and fractional excretion of sodium was more than 3%. No urinary eosinophils were detected. Antinuclear antibodies showed a positive nucleolar pattern titer (1:320). Cytoplasmic antineutrophil cytoplasmic antibodies, perinuclear antineutrophil cytoplasmic antibodies, anti-dsDNA antibodies, anti-Ro antibodies and anti-Sm antibodies were all negative, with normal complement component 3 and complement component 4 levels. Urine immunofixation showed no monoclonal protein in the urine. The patient presented hyperchloremic high anion gap metabolic acidosis, which improved after intravenous administration of sodium bicarbonate. During admission she was assessed by the Hematology Department because of increased urine albumin-to-creatinine ratio and serum monoclonal kappa and lambda light chains; however, monoclonal gammopathy was ultimately excluded. At subsequent follow-up by the Hematology Department the patient was diagnosed with pernicious anemia. A kidney biopsy revealed the presence of lymphoplasmacytic interstitial infiltrate. Positron emission tomography/computed tomography did not show viable tumor tissue. Although Doppler ultrasonography did not show abnormalities of renal vascular flow, it revealed cholelithiasis. Kidney ultrasound confirmed cholelithiasis and also showed nonobstructive dilatation of the collecting system of the right kidney, clinically insignificant.

The patient was treated with a blood transfusion and fluid repletion with isotonic saline and presented excellent improvement with corticosteroid treatment 50 mg daily, which was tapered over the next month. Her creatinine was stable at 1.5 mg/dL with estimated glomerular filtration rate 46 mL/min/1.73 m^2^. Drug etiology was suspected; omeprazole was replaced with famotidine and the patient was discharged and referred to the Pharmacology Department to assess the case.

The nephritis’ drug causality was assessed with the causality assessment in reports on adverse drug reactions algorithm of the SEFV (Aguirre et al., 2016). Omeprazole (score +5) was the drug involved according to the algorithm, metformine (score +2) was conditionals and the rest of medication (score ≤ 0) were not related. Seven months later, an LTT was performed to evaluate drugs with a related causality (SEFV score ≥ +4). The LTT did not show T cell proliferation with omeprazole, nor did flow cytometry. In contrast, both LTT and flow cytometry showed activation of T cells with PS 80. The patient was taking omeprazole-g, which contains PS 80 as an excipient (SI = 3 and SI = 5.6, respectively). Metformin also was tested and showed a negative result.

According to these results, we prohibited any medication that contained PS 80 as an excipient and recommended continuing with famotidine instead of omeprazole to avoid occasional dispensation of omeprazole from laboratories that use PS 80 as an excipient.

## Discussion

PEG and polysorbates are compounds that contain polyether groups. They are extensively used in medical and commercial applications, often as excipients in liquid and solid medication formulations. Allergic reactions, including anaphylaxis, have been reported in some cases of polyethylene glycol use during colonoscopy preparation (Wenande and Garvey, 2016). Although the mechanism behind PEG hypersensitivity is not well understood, type I hypersensitivity could be involved in clinical reactions to unconjugated PEGs (Stone et al., 2019).

The immune reaction triggered by drugs or their reactive by-products is considered a primary pathogenic mechanism in organ-specific hypersensitivity reactions to medications. Our assessment using the LTT revealed limitations, primarily influenced by the clinical condition and the specific drug causing the adverse reaction. The lack of standardization contributes to considerable variability in the accuracy across published studies (Mayorga et al., 2016). The LTT demonstrates an average sensitivity of 56% and specificity of 94%. Studies suggest its higher efficacy in diagnosing moderate delayed drug hypersensitivity reactions compared to severe organ-specific reactions (Mayorga et al., 2017). In DILI, LTT showed a specificity of 100% but a lower and a sensitivity of 77% (Rodri et al., 2022).

Causality assessment was conducted using the SEFV algorithm (Aguirre et al., 2016) and updated RUCAM for the hepatitis case (Danan and Teschke, 2016). However, the SEVF algorithm exhibits limitations in accurately distinguishing between closely related categories such as “possible” and “probable.” Moreover, it has restricted applicability in cases leading to fatal outcomes. Similarly, the RUCAM algorithm has limitations, including the inability to differentiate between concomitant hepatotoxic drugs with identical temporal sequences (García-Corte et al., 2011; Weber et al., 2021). Despite their utility, these algorithms cannot precisely quantify the likelihood of a connection, nor can they conclusively establish or dismiss causation or evaluate a drug’s role in the emergence of an adverse reaction (The Uppsala Monitoring Center; Doherty, 2009; Meyboom et al., 1997).

Drug-related AP is a rare condition that can be difficult for physicians to diagnose (Simons-Linares et al., 2019). Familiarity with the drugs that have the potential to cause this condition could help clinicians recognize this uncommon etiology, allowing them to prevent rechallenge of the offending medication and avoid harm to the patient. Although drug-induced pancreatitis has not been linked to PS 80 in the literature, we have only encountered 1 case of PEG-induced pancreatitis during bowel preparation for colonoscopy (Franga and Harris, 2000). Clinicians should also be aware of all other possible non-drug causes of acute pancreatitis when conducting formal causality assessments (Badalov et al., 2007).

In the case of our patient, this is the first case described in the literature of acute pancreatitis related to PS 80. We suggested the drug etiology after other non-drug causes had been excluded. We also took into consideration previous exposure to the excipient in the past.

DILI, characterized by hepatic or biliary system damage resulting from the consumption of hepatotoxic drugs, is the prevailing etiology of acute liver failure in Western societies (Bjo et al., 2022). The pathogenesis of DILI is intricate, involving a multifactorial interplay of genetic, metabolic and immunological elements (Kuna et al., 2018). Hepatotoxicity associated with the use of the AMX-CLV combination is well established in the literature (Ersoz et al., 2001; Gresser, 2001). This relationship appears to be more closely related to clavulanic acid or its interaction with amoxicillin, given that the frequency of adverse reactions is significantly higher with the combination of both drugs than with the use of amoxicillin alone, although cross-reaction can occur (Mari et al., 2000). The latency period for hepatotoxicity can vary widely, ranging from 2–45 days, which can greatly hinder diagnosis (Jordán et al., 2002; Borja et al., 2004). Several studies have identified AMX-CLV as the most common drug associated with DILI (Andrade et al., 2005), whereas macrogols and polysorbate are not mentioned as causative agents in the literature (LiverTox, 2012).

As for case 2, we continued searching for the DILI’s etiology due to the negative LLT result. We believe that the origin of the liver damage was produced more likely by both medications, macrogols and AMX-CLV.

AIN is one of the leading causes of acute kidney injury, and it is believed that the actual frequency is probably underestimated. Etiology includes drug-induced, autoimmune, infectious and idiopathic forms. The most common AIN etiology is drug-induced disease, which is thought to underlie 60%–70% of cases. Among the drugs potentially causing AIN, antibiotics, non-steroidal anti-inflammatory drugs and proton pump inhibitors are the most frequent culprits (Perazella and Markowitz, 2010). Our case 3 is the first case described in the literature of AIN related to PS 80. We suggested the excipient etiology after other non-drug causes had been ruled out and taking into account the LTT results and analyzing CD69 expression with the active ingredient and PS 80.

Preliminary results from our group also indicate that LTT with PEG and polysorbates is a useful tool for identifying excipients as causal agents in delayed hypersensitivity reactions to COVID-19 vaccines, and it can play an important role in risk stratification in patients with hypersensitivity reactions (Ruiz-Ferna et al., 2023). The present cases indicate that a causality assessment in organ-specific immune-mediated reactions should consider the role played by excipients, especially in cases with drugs which scored a high causality but negative test results with the active ingredient and cases of hypersensitivity reactions to unrelated drugs sharing the same excipient.

## Patient pespectives

Allergy to excipients, such as PEG and polysorbates, should be suspected in patients with hypersensitivity reactions to an active ingredient manufactured with these excipients. LTT could be a useful tool to identify excipients as causal agents in delayed hypersensitivity reactions.

## Data Availability

The raw data supporting the conclusion of this article will be made available by the authors, without undue reservation.

## References

[B1] Aguirre C. Garcia M. (2016). Causality assessment in reports on adverse drug reactions. Algorithm of Spanish pharmacovigilance system. Med. Clin. Barc. 147 (10), 461–464. 10.1016/j.medcli.2016.06.012 27450163

[B2] Aithal G. P. Watkins P. B. Andrade R. J. Larrey D. Molokhia M. Takikawa H. (2011). Case definition and phenotype standardization in drug-induced liver injury. Clin. Pharmacol. Ther. 89 (6), 806–815. 10.1038/clpt.2011.58 21544079

[B3] Andrade R. J. Lucena M. I. Fernandez M. C. Pelaez G. Pachkoria K. Garcia-Ruiz E. (2005). Drug-induced liver injury: an analysis of 461 incidences submitted to the Spanish registry over a 10-year period. Gastroenterology 129 (2), 512–521. 10.1016/j.gastro.2005.05.006 16083708

[B4] Badalov N. Baradarian R. Iswara K. Li J. Steinberg W. Tenner S. (2007). Drug-induced acute pancreatitis: an evidence-based review. Clin Gastroenterol Hepatol Off Clin Pr. Am Gastroenterol Assoc. junio 5 (6), 648–661. 10.1016/j.cgh.2006.11.023 17395548

[B5] Beeler A. Zaccaria L. Kawabata T. Gerber B. O. Pichler W. J. (2008). CD69 upregulation on T cells as an *in vitro* marker for delayed-type drug hypersensitivity. Allergy 63 (2), 181–188. 10.1111/j.1398-9995.2007.01516.x 18005225

[B6] Bjornsson H. K. Bjornsson E. S. (2022). Drug-induced liver injury: pathogenesis, epidemiology, clinical features, and practical management. Eur. J. Intern Med. 97, 26–31. 10.1016/j.ejim.2021.10.035 34772600

[B7] Borja J. Rigau D. Souto M. (2004). Cholestatic hepatitis due to amoxicillin-clavulanic acid with positive re-exposure: importance of proper terminology in drug vigilance. Enferm. Infecc. Microbiol. Clin. 22 (1), 59. 10.1016/s0213-005x(04)73033-5 14757011

[B8] Danan G. Teschke R. (2016). RUCAM in drug and herb induced liver injury: the update. Int. J. Mol. Sci. 17 (1), 14. 10.3390/ijms17010014 PMC4730261 26712744

[B9] Doherty M. J. (2009). Algorithms for assessing the probability of an adverse drug reaction. Respir. Med. CME 2 (2), 63–67. 10.1016/j.rmedc.2009.01.004

[B10] Ersoz G. Karasu Z. Yildiz C. Akarca U. S. Yuce G. Batur Y. (2001). Severe toxic hepatitis associated with amoxycillin and clavulanic acid. J. Clin. Pharm. Ther. 26 (3), 225–229. 10.1046/j.1365-2710.2001.00341.x 11422607

[B11] European Medicine Agency Polysorbates. Available at: https://www.ema.europa.eu/en/polysorbates (Accessed November 28, 2023).

[B12] Franga D. L. Harris J. A. (2000). Polyethylene glycol-induced pancreatitis. Gastrointest Endosc. 1 diciembre 52 (6), 789–791. 10.1067/mge.2000.109718 11115922

[B13] García-Cortes M. Stephens C. Lucena M. I. Fernandez-Castañer A. Andrade R. J. (2011). Causality assessment methods in drug induced liver injury: strengths and weaknesses. J. Hepatol. 55 (3), 683–691. 10.1016/j.jhep.2011.02.007 21349301

[B14] Gresser U. (2001). Amoxicillin-clavulanic acid therapy may be associated with severe side effects -- review of the literature. Eur Res. 20 abril 6 (4), 139–149. 11309226

[B15] Jordán T. Gonzalez M. Casado M. Suarez J. F. Pulido F. Guerrero E. (2002). Amoxicillin-clavulanic acid induced hepatotoxicity with progression to cirrhosis. Gastroenterol. Hepatol. 25 (4), 240–243. 10.1016/s0210-5705(02)70252-5 11975871

[B16] Katarey D. Verma S. (2016). Drug-induced liver injury. Clin. Med. (Lond). 16 (Suppl. 6), s104–s109. 10.7861/clinmedicine.16-6-s104 27956449 PMC6329561

[B17] Kuna L. Bozic I. Kizivat T. Bojanic K. Mrso M. Kralj E. (2018). Models of drug induced liver injury (DILI) - current issues and future perspectives. Curr. Drug Metab. 19 (10), 830–838. 10.2174/1389200219666180523095355 29788883 PMC6174638

[B18] Leigh Ann Anderson P. Drug interaction checker. Available at: https://www.drugs.com/drug_interactions.html.

[B19] LiverTox (2012). Clinical and research information on drug-induced liver injury. Bethesda (MD): National Institute of Diabetes and Digestive and Kidney Diseases. Available at: http://www.ncbi.nlm.nih.gov/books/NBK547852/.

[B20] Macedo A. F. Marques F. B. Ribeiro C. F. (2006). Can decisional algorithms replace global introspection in the individual causality assessment of spontaneously reported ADRs? Drug Saf. 29 (8), 697–702. 10.2165/00002018-200629080-00006 16872243

[B21] Mari J. Y. Guy C. Beyens M. N. Ollagnier M. (2000). Delayed drug-induced hepatic injury. Evoking the role of amoxicillin-clavulinic acid combination. Therapie 55 (6), 699–704. 11234465

[B22] Mayorga C. Celik G. Rouzaire P. Whitaker P. Bonadonna P. Rodrigues-Cernadas J. (2016). *In vitro* tests for drug hypersensitivity reactions: an ENDA/EAACI Drug Allergy Interest Group position paper. Allergy 71 (8), 1103–1134. 10.1111/all.12886 26991315

[B23] Mayorga C. Dona I. Perez-Inestrosa E. Fernandez T. D. Torres M. J. (2017). The value of *in vitro* tests to DiminishDrug challenges. Int. J. Mol. Sci. 18 (6), 1222. 10.3390/ijms18061222 28590437 PMC5486045

[B24] Meyboom R. H. Hekster Y. A. Egberts A. C. Gribnau F. W. Edwards I. R. (1997). Causal or casual? The role of causality assessment in pharmacovigilance. Drug Saf. 17 (6), 374–389. 10.2165/00002018-199717060-00004 9429837

[B25] Perazella M. A. Markowitz G. S. (2010). Drug-induced acute interstitial nephritis. Nat. Rev. Nephrol. 6 (8), 461–470. 10.1038/nrneph.2010.71 20517290

[B26] Rodriguez A. Garcia-García I. Martinez de Soto L. Gómez Lopez De Las Huertas A. Borobia A. M. Gonzalez-Torbay A. (2022). Utility of lymphocyte transformation test for assisting updated Roussel Uclaf causality assessment method in drug-induced liver injury: a case-control study. Front. Pharmacol. 13, 819589. 10.3389/fphar.2022.819589 35370653 PMC8965575

[B27] Ruiz-Fernandez C. Cuesta R. Martín-Lopez S. Guijarro J. Lopez Gomez de Las Huertas A. Urroz M. (2023). Immune-mediated organ-specific reactions to COVID-19 vaccines: a retrospective descriptive study. Pharm. (Basel) 16 (5), 720. 10.3390/ph16050720 PMC10222899 37242502

[B28] Sanchez-Alamo B. Cases-Corona C. Fernandez-Juarez G. (2023). Facing the challenge of drug-induced acute interstitial nephritis. Nephron 147 (2), 78–90. 10.1159/000525561 35830831

[B29] Simons-Linares C. R. Elkhouly M. A. Salazar M. J. (2019). Drug-induced acute pancreatitis in adults: an update. diciembre 48 (10), 1263–1273. 10.1097/MPA.0000000000001428 31688589

[B30] Stone C. A. Jr Liu Y. Relling M. V. Krantz M. S. Pratt A. L. Abreo A. (2019). Immediate hypersensitivity to polyethylene glycols and polysorbates: more common than we have recognized. J. Allergy Clin. Immunol. Pract. 7 (5), 1533–1540. 10.1016/j.jaip.2018.12.003 30557713 PMC6706272

[B31] The Uppsala Monitoring Center The use of the WHO-UMC system for standardised case causality assessment. Available at: https://www.who.int/docs/default-source/medicines/pharmacovigilance/whocausality-assessment.pdf (Accessed November 24, 2023).

[B32] Weber S. Benesic A. Neumann J. Gerbes A. L. (2021). Liver injury associated with metamizole exposure: features of an underestimated adverse event. Drug Saf. 44 (6), 669–680. 10.1007/s40264-021-01049-z 33638811 PMC8184550

[B33] Wenande E. Garvey L. H. (2016). Immediate-type hypersensitivity to polyethylene glycols: a review. Clin. Exp. Allergy 46 (7), 907–922. 10.1111/cea.12760 27196817

[B34] Wolfe D. Kanji S. Yazdi F. Barbeau P. Rice D. Beck A. (2020). Drug induced pancreatitis: a systematic review of case reports to determine potential drug associations. PLoS One 15 (4), e0231883. 10.1371/journal.pone.0231883 32302358 PMC7164626

[B35] Yin L. Pang Y. Shan L. Gu J. (2022). The *in vivo* pharmacokinetics of block copolymers containing polyethylene glycol used in nanocarrier drug delivery systems. Drug Metab. Dispos. 50 (6), 827–836. 10.1124/dmd.121.000568 35066464

