# Supplementary material for: Organ-specific immune-mediated reactions to polyethylene glycol and polysorbate excipients: three case reports
Source: Front Pharmacol. 2024 Jan 3;14:1293294. doi: 10.3389/fphar.2023.1293294 (PMC10792031; doi:10.3389/fphar.2023.1293294)
Supplement: Supplementary file 1 [file DataSheet1.DOCX]

# TITLE: Organ-specific immune-mediated reactions to polyethylene glycol and polysorbate excipients: three case reports

**AUTHORS:** Olga Rogozina^1^, Carmen Ruiz-Fernández^2^, Susana Martín-López^1^, Ibtissam Akatbach-Bousaid^2^, Miguel González-Muñoz^2*^, Elena Ramírez^1*^

**Affiliations:**

**^1^** Clinical Pharmacology Department, La Paz University Hospital-IdiPAZ, Faculty of Medicine, Autonomous University of Madrid, 28046 Madrid, Spain; [olga.rogozina@salud.madrid.org](mailto:olga.rogozina@salud.madrid.org) (O.G.); smartinlopez@salud.madrid.org (S.M.-L), [elena.ramirezg@uam.es](mailto:elena.ramirezg@uam.es) (E.R.)

^2^ Immunology Department, La Paz University Hospital-IdiPAZ, 28046 Madrid, Spain; carmen.ruiz@idipaz.es (C.R.-F.); [ibtissam.](mailto:ibtissam.)akatbach@salud.madrid.org (I.A.-B.) [miguel.gonzalez.munoz@salud.madrid.org](mailto:miguel.gonzalez.munoz@salud.madrid.org) (M.G.-M.)

^*^ Correspondence: [miguel.gonzalez.munoz@salud.madrid.org](mailto:miguel.gonzalez.munoz@salud.madrid.org) (M.G.-M.); [elena.ramirezg@uam.es](mailto:elena.ramirezg@uam.es) (E.R.); Tel./Fax: +34-917-277-558 (M.G.-M.); +34-917-277-559 (E.R.)

**Supplementary Methodology**

**Flow cytometric analysis**

Heparinized blood was drawn and samples were processed within 2 h of venipuncture. Blood was diluted ½ with RPMI 1640 medium and 100 µL aliquots were incubated in duplicate with 50 µL of several concentrations of drugs for 72 hours at 37^o^C in 5% CO_2_. Negative and positive controls were included by incubating the blood with or without PHA (final concentration 20 µg/mL), respectively. The following final drug concentrations were assayed: 500 and 200 µg/mL for amoxicillin, 100 and 10 µg/mL for omeprazole and 10 and 1 µg/mL for polysorbate 80 and PEG 3350.

To analyse T cells activation, cells were stained with CD3(IgG1κ, CloneUCHT1)-FITC/CD4(IgG2bκ, clone OKT4)-PerCP/CD69(IgG1κ, clone FN50)-PE or IgG1κ isotype control-PE (Biolegend, Barcelona, Spain) for 20 minutes in the dark. Then, samples were incubated with 2 mL of Lysing/Fixation Solution (Beckman Coulter, Madrid, Spain) for 15 min at room temperature. After centrifugation (400 g, 5 min) and washing with PBS, cells were resuspended with 250 µL of PBS and acquired in a Dxflex flow cytometer (Beckman Coulter). Lymphocyte population was gated using side scattering (SSC) and forward scattering (FSC) to exclude debris and dead cells. T cells were identified in a SSC/CD3 dot plot and, subsequently, CD4+ T cells were identified in a CD3/CD4 dot plot. At least 20,000 CD3+ cells were analysed. A stimulation index (SI) was calculated as the ratio of percentage of CD3+CD4+CD69+ cells in drug-stimulated cultures to baseline percentage in unstimulated cells. The result was considered positive if the SI was higher than 2. Flow cytometric analyses in three exposed healthy controls for each drug assayed were also performed and negative results (SI<2) were obtained.

**Lymphocyte transformation test**

The LTT was performed using different concentrations of the drug/drugs involved. Mononuclear cells were separated over a density gradient (Histopaque-1077, Sigma-Aldrich) from fresh peripheral blood and were incubated for 6 days at 10^6^ cells/ml with various drug concentrations in triplicate. The following final drug concentrations were assayed: 500, 200, 100 and 50 µg/mL for amoxicillin, 100, 10, 1 and 0.1 µg/mL for omeprazole and 10, 1, 0.1 and 0.01 µg/mL for polysorbate 80 and PEG 3350. Phytohemagglutinin (5 µg/ml) was used as a positive control. For the final 18 hours of the incubation period, proliferation was determined by the addition of [3H] thymidine (0.5 µCi/well). Proliferative responses were calculated as SI, defined as the ratio between the mean values of counts per minute in cultures with drug and those obtained without drug. A positive response was defined as an SI ≥2. LTT was performed in at least three exposed healthy controls for each drug and negative results (SI<2) were obtained.

**Spanish pharmacovigilance system algorithm**

| Algorithm of the Spanish Pharmacovigilance System |
| --- |
| **1. The chronology referred to as the interval between drug administration and effect :** |
| 1. Compatible (score +2) |
| 2. Not totally compatible (+1) |
| 3. No information (0) |
| 4. Incompatible chronology (-1) |
| 5. Particular case of syndrome of abstinence (+2). |
| **2. The literature, defining the degree of knowledge of the relationship between the drug and the effect:** |
| 1. Known in the literature of reference (+2): collected in Summary of Product Characteristics or in books of reference (Martindale, Meyer’s) . |
| 2. Occasionally known (+1): only found in published cases reports. |
| 3. Unknown (0) |
| 4. There is pharmacological information against relationship between medicine and the adverse effect (-1) |
| 3**. The evaluation of drug withdrawal:** |
| 1. Improves with the withdrawal (+2) |
| 2. Does not improve with withdrawal (-2) |
| 3. No improvement and no withdrawal (+1) |
| 4. Improves and there is no withdrawal (-2) |
| 5. No information (0) |
| 6. Death or irreversible effect (0) |
| 7. Improves by development of tolerance, to despite not to withdraw (+1) |
| 8. Improves with symptomatic treatment to despite not to withdraw (+1) |
| **4. The rechallenge effect:** |
| 1. Positive effect reappearance (+3) |
| 2. Negative, the effect does not reappear (-1) |
| 3. No re-exposure or no information (0) |
| 4. Death or irreversible effect (0) |
| 5. Positive for a different specialty with the same active ingredient or parent drug (+1) |
| **5. Alternative causes:** |
| 1. Yes, an illness or other drugs (-3). |
| 2. Similar likelihood for drug and other causes (-1) |
| 3. Missing information (0) |
| 4. There is not any alternative cause (+1) |
| **6. Contributing factors:** |
| 1. Yes (+1). |
| 2. No (0) |
| **7. Complementary explorations:** |
| 1. Yes (+1). |
| 2. No (0) |
| **Categories according to final score:**   - Not classified (lack of data)/Improbable: <0 - Conditional: 1-3 - Possible: 4-5 - Probable: 6-7 - Defined: ≥ 8 |
